# Exploring Immune Responses to SARS-CoV-2: Insights from Sinopharm (BBIBP-CorV)-Vaccinated Individuals in a Group of Venezuelan Admixed Volunteers

**DOI:** 10.3390/biomedicines13071550

**Published:** 2025-06-25

**Authors:** Alexis Hipólito García, Soriuska José Mayora, Christian Medina, Inírida Amada Belisario, Wendy Yaqueline Martínez, Francis Isamarg Crespo, Juan Bautista De Sanctis

**Affiliations:** 1 Institute of Immunology Nicolás Enrique Bianco, Faculty of Medicine, Universidad Central de Venezuela, Los Chaguaramos, Caracas 1040, Venezuela; soriuskamayora@gmail.com (S.J.M.); cmedima1108@gmail.com (C.M.); ibelisariogomez@gmail.com (I.A.B.); wendymartinez3003@gmail.com (W.Y.M.); drafranciscrespo@gmail.com (F.I.C.); 2 Institute of Molecular and Translational Medicine, Faculty of Medicine and Dentistry, Palacky University in Olomouc, Hnevotinska 1333/5, 77900 Olomouc, Czech Republic; 3 Czech Advanced Technology and Research Institute, Palacky University in Olomouc, Hnevotinska 1333/5, 77900 Olomouc, Czech Republic

**Keywords:** cellular response, SARS-CoV-2, inactivated virus, Sinopharm/BBIBP, IFN-γ, granzyme B

## Abstract

**Background**: Vaccines are crucial for preventing infectious diseases, as both humoral and cellular immune responses play a vital role in combating viral infections. The cellular immune response is crucial against SARS-CoV-2, particularly with the emergence of new variants that evade antibody neutralization. This study focuses on the immune memory response in individuals who have been vaccinated with the Sinopharm BBIBP-CorV vaccine. **Methods**: A cross-sectional study evaluated lymphocyte subpopulations using flow cytometry in 52 vaccinated adults (30 females, 22 males) who had been exposed to SARS-CoV-2 or diagnosed with COVID-19. Conducted from February to June 2023 during the Omicron variant’s circulation, this study assessed antigens—CD154 in CD4+ T cells, CD107 and CD314 in CD8+ T cells, CD314 in NK cells, and CD86 in CD19 B cells—after stimulation with viral peptides and an inactivated virus. Granzyme B and IFN-γ were quantified using ELISA. **Results**: The memory response, regardless of gender, age, or Body Mass Index (BMI), was mild but significant upon exposure to a viral antigen or inactivated virus. An increase in the secretion of IFN-γ and granzyme B was also observed. **Conclusions**: It is suggested that the vaccine was able to generate a mild long-term memory against the SARS-CoV-2 virus in vaccinated adult individuals, independent of gender and BMI.

## 1. Introduction

Viruses are infectious agents that contain genetic material, either RNA or DNA, that require a host for replication and can cause disease. While viral infections in humans are rarely fatal, mortality often occurs when viruses cross species barriers or when the immune system is weakened. Defenses against viruses typically involve various immune components, and the effectiveness of these mechanisms varies based on how specific viruses enter, replicate, and spread within the host [1,2].

COVID-19, caused by the SARS-CoV-2 virus, was first reported in December 2019 in Wuhan, China. It is part of a larger family of coronaviruses. The virus evolves through mutations, resulting in new variants [2]. Upon SARS-CoV-2 infection, the innate immune response is activated in host cells via pathogen recognition receptors (PRRs) that detect viral components. This activation leads to the expression of virus-stimulated genes (VSGs) through transcription factors like IRF3 and NF-κB, releasing type I and III interferons (IFNs). These IFNs create an antiviral state by inducing interferon-stimulated genes (ISGs) in neighboring cells and signal through the Jak-STAT pathway. The dysregulation of IFN responses, influenced by factors such as the viral protein ORF9 b and age, may increase susceptibility to SARS-CoV-2, especially in the elderly [3,4,5,6,7].

Investigating adaptive immunity in the context of SARS-CoV-2 infection and vaccination is essential for understanding COVID-19 [3,4,5,6,7]. Studies have highlighted the roles of CD4+ and CD8+ T lymphocytes, B cells, and neutralizing antibodies [8,9,10]. Still, other cell populations like monocytes, natural killer (NK) cells, mucosal T cells (MAIT), follicular cells, and T gamma delta (Tγδ) cells also play essential roles in infection and vaccination [11]. These cells may significantly influence COVID-19 vaccination strategies and the development of immune memory and trained immunity [12].

Recent advancements in quantifying antigen-specific T cell responses have increasingly utilized MHC class I and II tetramers to visualize the responses of CD8+ and CD4+ T lymphocytes, respectively [13,14]. Research using MHC class I tetramers has demonstrated that many activated CD8+ T lymphocytes during viral infections are virus-specific [13,14]. CD8 cytotoxic T lymphocytes (CTLs) recognize and eliminate virus-infected cells through perforin and granzymes or by engaging with the Fas ligand on target lymphocytes, thereby inducing apoptosis [14]. CD8+ T lymphocytes secrete cytokines such as IFN-γ and TNF-α, and their activation is regulated [14,15]. Normal T cells express and secrete RANTES, which exerts antiviral effects without necessarily leading to the death of the infected cells [15].

CD8+ T lymphocyte responses against SARS-CoV-2 include recognizing the S, M, and N proteins; nsp6; and ORF3a [9,15,16]. Memory CD8+ T cells circulate 20–50 days post-symptom onset and have a half-life of 225 days [17]. Most are CD45RA (TEMRA), which is crucial for protecting against severe disease. The rest of the cells are effector memory (TEM) and CD8 naïve central memory (TCM) [18]. TEMRA protects against severe disease, as shown in other viral infections [19]. Virus-specific CD8+ T lymphocytes with cytolytic capacity can be detected as early as day 1 post-symptom onset, peaking around 2 weeks [20]. Their rapid induction and ability to produce effector molecules like IFN-γ and granzyme B are linked to better COVID-19 outcomes [3,4,17,19].

On the other hand, a group of CD8 cells expresses the CD314 (NKG2D) receptor, which is involved in the antiviral response [20]. However, its activation is independent of antigens and depends on the expression of stress receptors, which serve as ligands for NKG2D, including MICA/B and ULBP. These bystander cells can aid in the elimination of infected cells that express those markers. The expression of CD314 is also essential for NK anti-viral and cytotoxic responses [21].

In Venezuela, the pandemic began on March 13, 2020, with cases rising significantly by mid-May. A national vaccination campaign started on February 19, 2021, using Sputnik V and Sinopharm BBIBP-CorV vaccines [22,23]. Most of the population was vaccinated with the BBIBP-CorV vaccine.

The BBIBP-CorV vaccine, an inactivated virus vaccine introduced in 2020, uses β-propiolactone for inactivation and aluminum hydroxide as an adjuvant [24,25]. Widely used globally, it induces both humoral and cellular immune responses. However, most studies focus on the humoral aspect, leaving a gap in understanding its impact on T cell, B cell, and NK cell memory [26]. The vaccination schedule is 0/21–28 days to prevent COVID-19. A study by Jara et al. reported efficacy rates of 65.9% against infection, 87.5% for hospitalizations, and 90.3% for critical admissions [26,27,28]. Low seroconversion may limit single-dose effectiveness; therefore, multiple vaccinations are advised [26,27,28]. The vaccine demonstrates partial effectiveness against various variants, including Omicron [29].

The immunological landscape of Latin American populations, especially in genetically admixed groups, is underexplored. Extensive vaccine coverage and natural SARS-CoV-2 exposure in Venezuela may lead to unique immune responses. Our study (February to June 2023, during the Omicron circulation) found that most individuals had been vaccinated or infected, creating a unique opportunity to investigate how pre-existing immune memory affects cellular responses to BBIBP-CorV.

This study aims to bridge the knowledge gap by characterizing the long-term cellular immune memory response induced by the Sinopharm/BBIBP-CorV vaccine in a cohort of 52 admixed Venezuelan adults. The activation markers on T lymphocytes were assessed (including CD154, CD107a, and CD314), as well as B lymphocytes (CD86) and NK cells. The measurement of IFN-γ and granzyme B was performed after in vitro stimulation with SARS-CoV-2-specific peptides and heat-inactivated virus. The goal is to present a more complete picture of the vaccine-induced immune landscape, relate these findings to natural immune responses during the Omicron wave, and ultimately assist in shaping future vaccination strategies for similar populations.

## 2. Materials and Methods

### 2.1. Characteristics of the Volunteers

This research study was conducted between February 2023 and June 2023. On 15 February 2023, the Ethical Committee of the Institute of Immunology at the Faculty of Medicine of the Central University of Venezuela approved the study, code 001/2023. Before undergoing heparin-anticoagulated venous sampling, the recruited volunteers were required to read and sign an informed consent form, entrusting the Ethical Committee with oversight of the generated data. Additionally, the Ethical Committee authorized the submission of the manuscript associated with this study.

The following inclusion criteria were established for the study: (1) the completion of the basic vaccination regimen of two doses of BBIBP-CorV vaccine [23,24,25,26]; (2) the absence of any recent infectious diseases at the time of sample collection, including a negative SARS-CoV-2 antigen test; (3) no history of autoimmune diseases as determined through laboratory screening (C-reactive protein, sedimentation rate, antinuclear antibody, rheumatoid factor, and anti-CCP); (4) no administration of immunosuppressive treatment; (5) participation from individuals of a genetically admixed Venezuelan population, as verified in our laboratory; and (6) adult individuals. Pregnant women, individuals who tested positive for the SARS-CoV-2 antigen, those from diverse genetic backgrounds, and those who had received alternative vaccines were excluded from participation. The process involved a detailed anamnesis, including in-depth questioning about any personal or family history of autoimmune disorders and response to infectious diseases. Participants with clinical or familial indicators suggestive of autoimmune disease or impaired immune response (immune deficiency) were excluded from the study.

The questionnaire also included details of the last three months before the sample collection. Three months before the sample collection, none of the patients suffered from SARS-CoV-2 infection or other respiratory infections.

A total of 52 individuals, comprising 30 females and 22 males, were successfully recruited for the study. The individuals did not suffer from a recent (less than 3 months) infection by SARS-CoV-2. In the previous years, most individuals (58%) documented moderate SARS-CoV-2 infection, and 42% had a mild illness. They were highly exposed to the virus and were either healthcare or service personnel.

### 2.2. Screening of Anti-RGD S Protein

The antibodies against the S protein’s receptor-binding domain (RBD) were analyzed using the commercial LEGEND MAX™ Spike SARS-CoV-2 (RBD) kit from BioLegend (San Diego, CA, USA), following the manufacturer’s instructions. The kit was previously validated at our Institution [30].

### 2.3. Stimulation of the Samples

**Figure 1:**
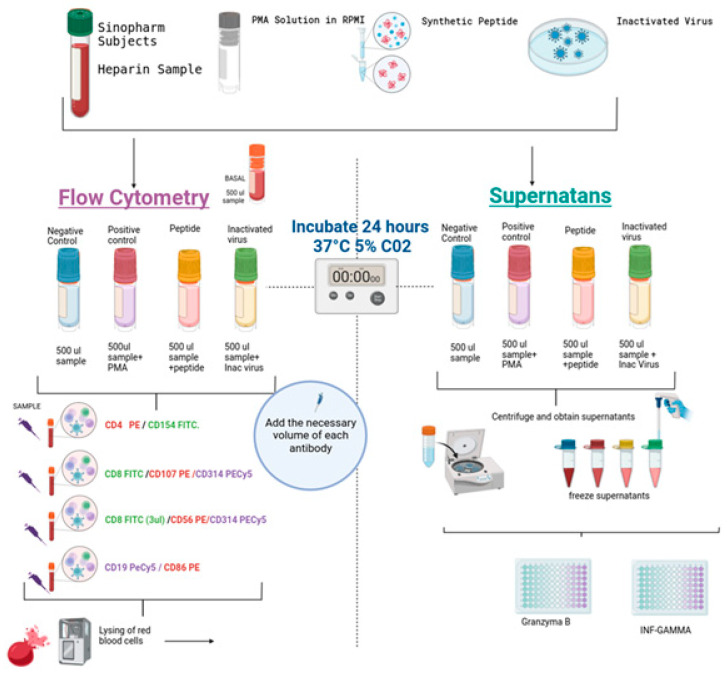
The methodologies employed for cell stimulation and subsequent analysis, along with the quantification of cytokines, utilize commercially available ELISA kits. The color represents different treatments: control magenta, positive control red, viral peptides in yellow and inactivated virus in green. The figure was made using software from Biorender.com (accessed on 15 March 2025).

Cell stimulation specific to SARS-CoV-2 was conducted to illustrate alterations in the expression of various activation markers on B cells, T cells (CD4 and CD8), and natural killer (NK) cells. Additionally, the analysis included the production of granzyme B and interferon-gamma (IFN-γ), as depicted in Figure 1. Four tubes per individual were used, as illustrated in Figure 1. The assay was performed in duplicate. A volume of 0.5 mL of blood was seeded in 24-well plates (Corning). One well was a control; the second was stimulated with SARS-CoV-2-specific synthetic peptides (PepPool: SARS-CoV-2 (SNMO), human code: 3622-1) from MABTECH (Nacka Strand, Sweden); the third was stimulated with 10 plaque-forming units of heat-inactivated virus (Wuhan Strain) obtained from the supernatants of Vero cell cultures, and its genetic integrity was verified by PCR. As a positive control of the assay, cells were stimulated with Cell Activation Cocktail (without brefeldin A) from BioLegend (cat. no. 423302 San Diego, CA, USA). Plates were assembled in duplicate and incubated at 37 °C in a 5% CO_2_ atmosphere for 24 h. Lymphocytes without stimulation were used as the negative control.

### 2.4. Expression of Activation Markers in Different Lymphocyte Populations and Subpopulations

After incubation, as shown in Figure 1, the cells were transferred to 12 × 75 mm tubes, and the samples were washed and labeled with the specific antibodies. Then, the erythrocytes were lysed with an automatic lyser (Beckman Coulter, Brea, CA, USA), and the samples were analyzed with flow cytometry using the Epics XL equipment from Beckman Coulter. The assay was performed in duplicate. The following panels of antibodies were used: (1) T-helper T-lymphocytes (CD4PE/CD154 FITC), (2) cytotoxic T-lymphocyte degranulation (CD8 FITC/CD107aPE), (3) bystander CD8 lymphocytes and NK cells (CD8 FITC/CD56 PE/CD314 PECY5), and (4) B lymphocytes (CD19 PECY5/CD86 PE). All antibodies used were obtained from BioLegend, and gating was performed on the lymphocyte population, with a minimum of 5000 events per sample.

It is important to note that all the samples responded to the positive stimulus (PMA/ionomycin) as expected.

### 2.5. IFNγ and Granzyme B ELISA

**Figure 2:**
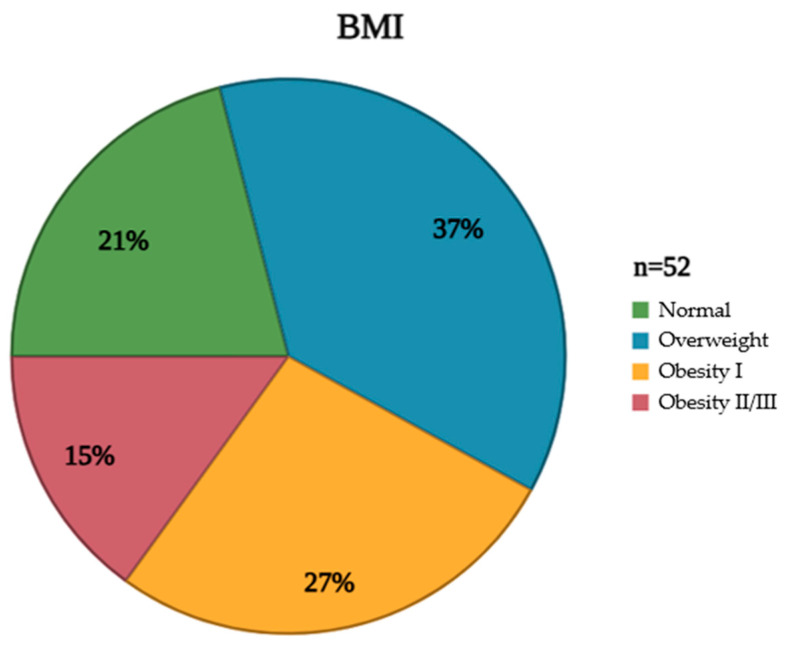
Illustrates the distribution of patients classified by Body Mass Index (BMI) categories according to the standards established by the World Health Organization. BMI: Body Mass Index. This figure was created utilizing the software from Biorender.com (accessed on 15 March 2025).

The supernatants of the stimulated cells were stored at −20 °C until use. IFN-γ and granzyme B were measured in triplicate using a sandwich ELISA from BioLegend, following the manufacturer’s recommended methodology. The minimum detectable concentration for the LEGEND MAX™ Human IFN-γ ELISA kit was 5.6 pg/mL. In contrast, the minimum detectable concentration for the LEGEND MAX™ Human granzyme B ELISA kit was 2.4 ± 1.2 pg/mL. The concentration of the cytokines was determined using a standard curve, and all diluted samples fell within the limits of the kit. As shown in Figure 2, the positive control for the cellular assays also served as the positive control for the ELISA.

### 2.6. Statistical Analysis

GraphPad Prism version 6 program was used for the statistical analysis. The comparison among groups was performed using ANOVA with Bonferroni corrections for the different groups. In specific cases, paired and unpaired Student’s *t*-tests were used. Pearson correlations were performed with different parameters, and significance was assessed in each case. Significance was considered when *p* < 0.05.

The number of samples required for the analysis was based on the following formula:

$$n=\frac{Z\alpha^{2}\times p_{0}\times q_{0}}{D^{2}}$$

The value of Z is 1.96 for a significance of *p* = 0.05; the p_0_ is 0.1; the q_0_ is 0.8.

Thus, the minimum assessment is 30 samples. The statistical analysis of the negative control vs. stimulated (positive control, viral peptides, or an inactivated virus) was performed using a paired Student’s *t*-test. Positive controls were always >5-fold the value of negative controls for each individual.

## 3. Results

The studied population exhibited diverse characteristics that were meticulously documented to understand the cohort’s composition comprehensively. These attributes, encompassing demographic, clinical, and anthropometric data, are crucial for contextualizing the subsequent analyses and interpreting the study’s findings. Furthermore, an assessment of clinical characteristics revealed the prevalence of various comorbidities, including hypertension and diabetes mellitus, providing insight into the overall health status of the population (Table 1). In addition to these clinical parameters, BMI distribution within the studied group was carefully evaluated and visually represented. This distribution, illustrating the frequency of individuals falling within different BMI categories (underweight, normal weight, overweight, and obese), provides a valuable perspective on the population’s weight status and its potential impact on the vaccine response (Figure 2).

Most cohort individuals had antibodies against the RBD of the Spike protein (70.5%) with values ≥ 40 IU/mL, while 29.5% had values lower than 40 IU/mL. From the entire cohort, 10.5% had values lower than 10 IU/mL but higher than 2 IU/mL, which is the cutoff value of the kit. These individuals with low titers received two doses of the vaccine.

**Figure 3:**
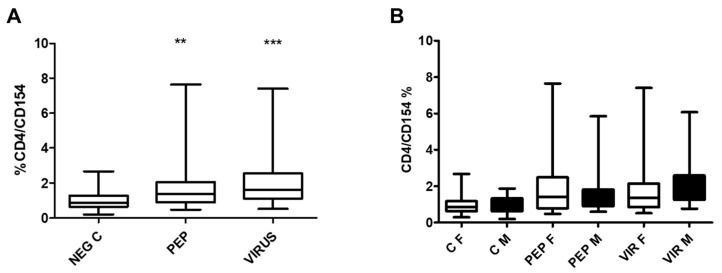
Effects of viral peptides and an inactivated virus on the activation of CD4+ T cells. (**A**) Overall impact observed in the cohort. The increased expression of CD154 was statistically significant (** *p* < 0.01 and *** *p* < 0.001, Bonferroni post hoc test) as determined by repeated measure ANOVA, with a sample size of 52 individuals analyzed in pairs. (**B**) Data were stratified by gender. Although no significant differences were identified between genders (30 females and 22 males) across the conditions, notable differences were established within each gender group when comparing negative controls to viral peptides, as well as negative controls to an inactivated virus (*p* < 0.01 in both scenarios). C denotes control, PEP signifies viral peptides, VIR represents inactivated virus, F indicates female, and M denotes male, NEG C refers to negative control.

Figure 3 illustrates the effect of peptides and an inactivated virus on the expression of CD154 in CD4 cells in the whole group (Figure 3A) and divided by gender (Figure 3B). Significant differences were obtained in stimulated cells as compared to the control. However, when the results were analyzed by gender (Figure 3B), no significant difference was found in the expression of the antigen CD154 in females vs. males. Significant differences were maintained when the stimulated groups were compared to the negative controls.

**Figure 4:**
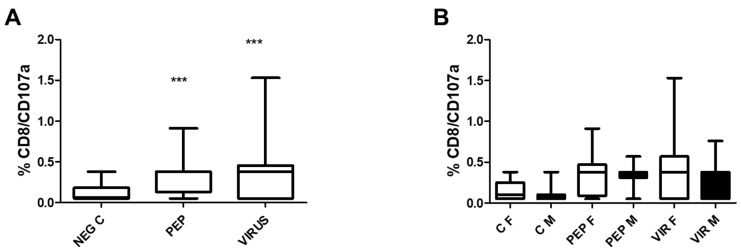
Effects of viral peptides and an inactivated virus on the degranulation of CD8+ T cells, as assessed by CD107a expression. (**A**) Results of the entire group, demonstrating a significant increase in CD107a expression (*** *p* < 0.001, Bonferroni post hoc test) as determined by repeated measure ANOVA, with 52 individuals included in the paired analysis. (**B**) Data are segregated by gender, revealing no significant differences between the genders (comprising 30 females and 22 males) across the various conditions. However, notable differences were identified within each gender when comparing the negative controls with viral peptides to the negative control with an inactivated virus (*p* < 0.01 for both comparisons). The abbreviations used are as follows: C for control, PEP for viral peptides, VIR for an inactivated virus, F for female, and M for male, NEG C refers to negative control.

Figure 4 illustrates the induction of degranulation in response to stimulation by CD8 T cells. Figure 4A represents the effect of the whole group, in which significant differences were observed when the stimuli were compared to the control. Despite the significance recorded in Figure 4A, there were no significant differences between genders in Figure 4B. Nevertheless, as recorded in the previous figure, significant differences were observed between the stimuli and the negative control.

**Figure 5:**
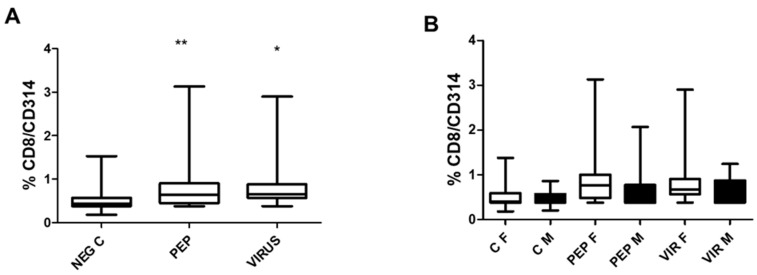
Impact of viral peptides and inactivated virus on the expression of CD314 (NKG2D). (**A**) Effect on the entire cohort. The increase in expression of CD314 was statistically significant (* *p* < 0.05, ** *p* < 0.001, Bonferroni post hoc test), as determined by repeated measure ANOVA with a sample size of *n* = 52 individuals in paired analysis. (**B**) Cohort was analyzed by gender. There were no significant differences between genders (30 females and 22 males) across each condition. Nonetheless, significant differences were identified within each gender when comparing negative controls to viral peptides and between negative controls and an inactivated virus (*p* < 0.01 in both instances). The notation includes C for control, PEP for viral peptides, VIR for inactivated viruses, F for females, and M for males, NEG C refers to negative control.

Figure 5 depicts the effect of peptides and an inactivated virus on the induction of CD314 (NKG2D) expression, representing T CD8 bystander cells. Both stimuli significantly increased CD314 expression, as shown in Figure 5A. In Figure 5B, the effect of gender is represented, which is similar to that observed for the other markers CD4/CD154 and CD8/CD107a. The specific response to each situation is identical in both genders.

**Figure 6:**
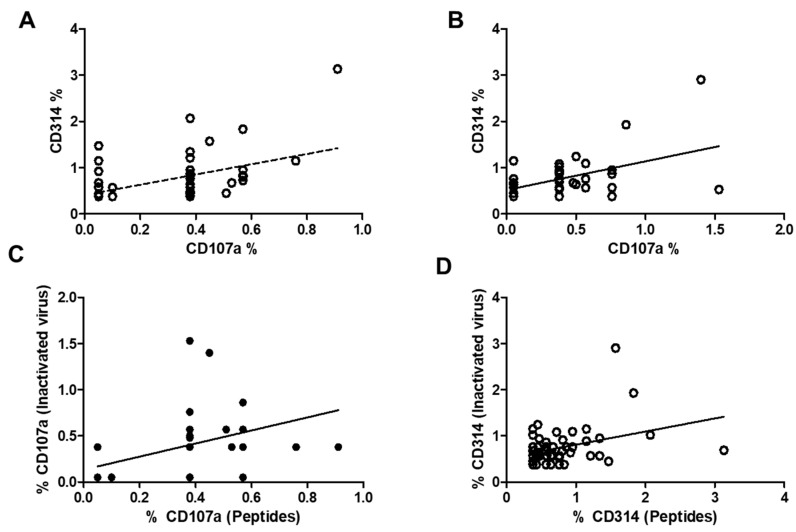
Correlation between the expression of CD314 and CD107a in stimulated samples. (**A**) Correlation is evaluated using viral peptides, yielding a correlation coefficient of r = 0.32 with a significance level of *p* < 0.01 (*n* = 52). (**B**) Correlation using an inactivated virus, demonstrating a stronger correlation (r = 0.47, *p* < 0.001). (**C**) Correlation between CD107a expression and both stimuli, which is statistically significant (r = 0.44, *p* < 0.005). (**D**) Correlation between the expression of CD314 and both stimuli is also illustrated, showing a marginally significant result (r = 0.35, *p* = 0.01).

Figure 6 illustrates the correlation between the expression of CD107a and CD314 in stimulated CD8 cells, memory CD8 cells, and bystander CD8 cells using viral peptides (Figure 6A) or an inactivated virus (Figure 6B), as well as the correlation between both markers in CD107a expression (Figure 6C) and CD314 expression (Figure 6D). Correlations were observed in all the graphs and are statistically significant. Figure 6C,D represents both correlations to address the difference in the increase in both markers depending on the stimulus. In Figure 6C, representing CD107a, it is clear that some individuals did not respond to an inactivated virus, with two individuals having very low responses to both stimuli, while others had a higher response. For the CD314 expression (Figure 6D), most values are concentrated in the lower part of the figure, suggesting similar expression post-stimulation; only four individuals differed from this response.

**Figure 7:**
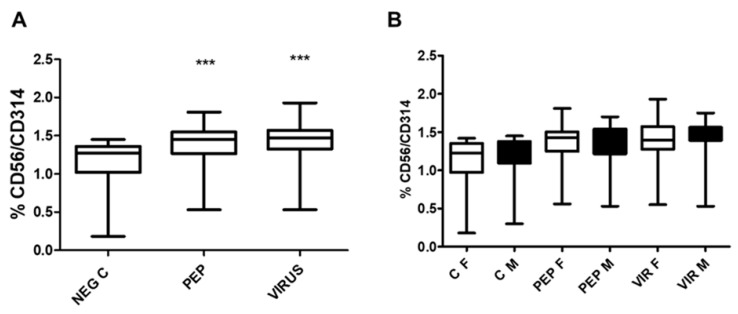
Effect of viral peptides and inactivated viruses on the expression of CD314 (NKG2D). (**A**) Overall impact on the entire study cohort. The observed increase in CD314 expression was statistically significant (*** *p* < 0.0001, Bonferroni post hoc test), as determined by repeated measure ANOVA, with a total of *n* = 52 individuals in paired analysis. (**B**) The group was stratified by gender. Although no significant differences were found between genders (comprising 30 females and 22 males) within each condition, significant disparities were identified within each gender when comparing negative controls with viral peptides to negative controls with an inactivated virus (*p* < 0.01 in both instances). To clarify the abbreviations used, C denotes control, PEP represents viral peptides, VIR indicates an inactivated virus, F refers to female, and M signifies male, NEG C refers to negative control.

The expression of the killing receptor CD314 was also assessed in NK cells, as illustrated in Figure 7, which shows an increase in CD314 expression upon activation by viral peptides and an inactivated virus (Figure 7A). As observed in CD8 cells for CD107a and CD314 cells, no differences were found when the groups were separated by gender (Figure 7B). However, a positive increase was recorded with peptides and an inactivated virus for each stimulus analyzed.

Even though the expression of CD314 in CD8+ T cells and NK cells increases upon stimulation with viral peptides or an inactivated virus, there is no correlation between the percentage of positivity observed for both cell types (r = 0.1 for peptides and r = 0.01 for an inactivated virus).

**Figure 8:**
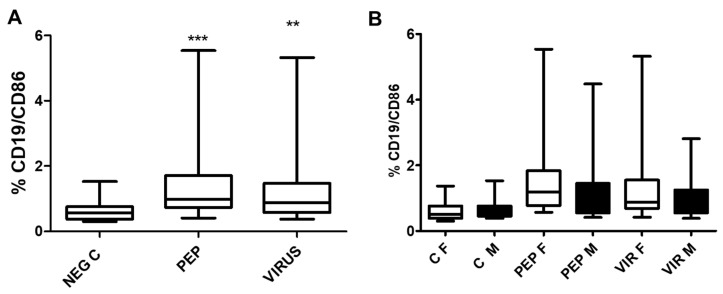
Analysis of the effect of viral peptides and an inactivated virus on the expression of CD86 in B lymphocytes. (**A**) Outcomes observed across the study population. The increase in CD86 expression was statistically significant (** *p* < 0.001, *** *p* < 0.0001, Bonferroni post hoc test), as determined through repeated measure ANOVA, with a sample size of *n* = 52 individuals in the paired analysis. (**B**) Data by gender, revealing no significant differences across the various conditions between the female (*n* = 30) and male (*n* = 22) groups. Nevertheless, significant differences were identified within each gender when comparing the negative controls to viral peptides and an inactivated virus, yielding a *p*-value of less than 0.01. Additionally, the notation used in the figure is defined as follows: C represents control, PEP denotes viral peptides, VIR indicates an inactivated virus, F represents female, and M indicates male, NEG C refers to negative control.

The effect of viral peptides and an inactivated virus on the expression of the B lymphocyte activation marker C86 in B cells is illustrated in Figure 8. The increase in expression is significant for both stimuli (Figure 8A). As observed with other markers, there is no statistical difference between genders (Figure 8B). However, the difference in stimulated cells versus control is significant (*p* < 0.01) for both stimulators.

**Figure 9:**
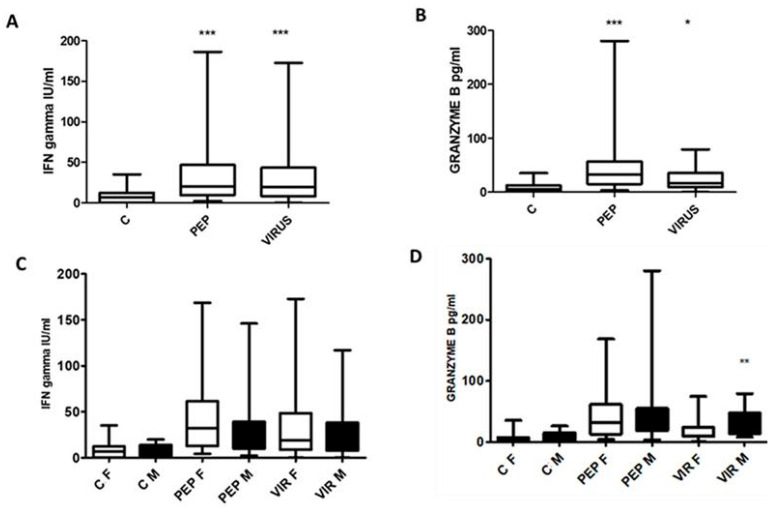
Interferon gamma (IFN-γ) levels (**A**) and granzyme B (**B**), following stimulation with peptides and an inactivated virus. Statistically significant differences were observed for both stimuli, with IFN-γ displaying significance at *** *p* < 0.001, and granzyme B showing significance at *** *p* < 0.001 and * *p* < 0.01. When analyzing the data by gender, no significant differences were noted in IFN-γ secretion (**C**); however, a notable increase in granzyme B was recorded among male participants stimulated with an inactivated virus (**D**), with significance at ** *p* < 0.005. To clarify the abbreviations used, C denotes control, PEP represents viral peptides, VIR indicates an inactivated virus, F refers to female, and M signifies male.

The secretion of IFN-γ and granzyme B in response to stimulation is illustrated in Figure 9A,B, and the effect of gender is shown in Figure 9C,D. Significant differences were observed in the secretion of the entire cohort; however, only male volunteers produced significantly more granzyme B than women in response to stimulation with an inactivated virus. Nonetheless, this difference in granzyme B secretion was not associated with any other parameter.

There is no significant correlation among the values obtained for IFNγ and granzyme B (r = 0.1), between the expression of CD154 and IFNγ (r = 0.05), and between granzyme B and CD8/CD107a (r = 0.2), CD8/CD314 (r = 0.12), and CD56/CD314 (r = 0.08).

There is no correlation between the titers of the RGD antibody and any of the cellular parameters analyzed or the granzyme B and IFN-γ values. Moreover, there was no significant difference when the group was separated by vaccine dose or COVID-19 complications in the different BMI groups (normal weight vs. overweight/obese); the statistical analysis revealed a *p*-value greater than 0.2 for each marker analyzed.

## 4. Discussion

The BBIBP-CorV vaccine is an inactivated vaccine made from cultured virus particles and then inactivated to stimulate an immune response without causing disease. It is produced using African green monkey kidney cells (Vero cells) inoculated with the SARS-CoV-2 WIV04 strain [2]. In Venezuela, during the study period, genomic surveillance by the Instituto Venezolano de Investigaciones Científicas (IVIC) and Instituto Nacional de Higiene (INH) confirmed that the Omicron variant was circulating, with 141 sequences submitted to the GISAID database, all belonging to this variant.

Although the Sinopharm vaccine has been shown to offer around 65% protection against the original strain, the vaccine has been shown to partially protect against other variants, including the Omicron variant [26,27,28]. Ongoing research examines the variant-specific response of T lymphocytes induced by Sinopharm against emerging SARS-CoV-2 variants. Some studies suggest that these T cell responses are more effective against the original Wuhan strain [31,32,33]. While T lymphocyte responses exist for newer variants, they are generally stronger against the Wuhan strain [34,35].

Research on COVID-19 vaccines has primarily focused on neutralizing antibodies, while the importance of T cell response for protection against severe COVID-19, especially with variants that evade antibody recognition, is gaining recognition [8,9,33]. The development of these vaccines has significantly changed the management of the SARS-CoV-2 pandemic [8,9,33]. Studies show that the Sinopharm vaccine induces a memory T cell response, protecting the initial antibody response [31]. Research by Ning J. and colleagues [32] indicates the activation of CD4+ and CD8+ T cells after vaccination, with CD4+ T cells coordinating the immune response and CD8+ T cells targeting infected cells [32]. However, there is limited analysis on B cell and NK cell activation and the role of CD8 bystander cells. Additionally, the vaccine’s response may vary by gender and obesity [36], similar to other vaccines, although this has not been thoroughly investigated in clinical trials.

The BBIBP-CorV vaccine stimulates a cellular immune response with T cell activation, reducing severe disease and mortality from the virus. Ma et al. [37] showed that plasma B lymphocytes produce antibodies against SARS-CoV-2 after vaccination, although these antibodies decrease over time. Tong et al. [38] studied nine unvaccinated healthy individuals (aged 27–66) and found an increase in monocytes, central memory CD4+ T lymphocytes, and memory B lymphocytes after vaccination. TCR-seq and RNA-seq analyses revealed the clonal expansion of CD4+ T lymphocytes post-booster vaccination, while TCR diversity among these cells decreased. This suggests that inactivated vaccines like BBIBP-CorV primarily induce a CD4+ T cell immune response.

In a recent review, Mortari et al. [39] compared different responses to vaccines in the European population, concluding that long-lasting memory B cell responses are observed in individuals vaccinated with heterologous vaccines. This conclusion, however, does include the possibility that infection with different virus variants after a homologous vaccination would produce effects similar to heterologous vaccination. It is possible that in countries where the BBIBP-CorV vaccine or similar inactivated viral vaccines are used, herd immunity may play a crucial role in maintaining a memory response compared to vaccines based solely on the viral spike protein. Additionally, the presence of other viral proteins, such as the immunogenic N protein, can be more effective in maintaining a memory antiviral response. The roles of various cell types, including bystander CD8, NK, NKT, and Tγδ cells, in the protective antiviral response induced by vaccines are still not well-defined.

The analysis conducted in this manuscript, utilizing commercial viral peptides and an inactivated virus, enhances our understanding of cellular responses upon activation. The observation that all markers were significantly upregulated across various cell populations and subpopulations underscores the extensive memory response following viral exposure. The findings presented in this current report reveal notable differences in cellular responses to viral peptides and inactivated viruses. While the existing literature has examined the cellular responses to mRNA and vector-based vaccines [31,32,33,34,35], direct comparisons of vaccine efficacy based solely on cellular reactions to viral proteins pose significant challenges, as analyzed in our recent review [36]. Longitudinal studies are deemed more suitable for assessing efficacy, as new viral infections may alter the immune response [36]. In this context, longitudinal studies have not provided substantial evidence regarding the influence of gender and BMI on several vaccines despite their effect on immune response [36]. In the case of COVID-19, multiple vaccinations and herd immunity may be responsible, in the long term, for a more sustainable memory immune response.

Notably, the activation of CD8 bystander cells (Figure 5A,B), which are independent of antigen recognition, illustrates the diversity of the immune response [19,20]. A significant correlation was identified between the expression of CD107a and CD314 in CD8 cells (Figure 6A–D), specifically in the context of viral stimulation. These findings introduce new perspectives regarding the importance of comprehensively analyzing the immune response, which many groups have not explored. Similarly, the activation of NK cells, as indicated by CD314, provides valuable insights into the role of cellular stress induced by viral contact, which serves as a crucial signal to activate the immune response.

Memory NK responses upon viral infection have been studied in several viral infections [40,41]; however, no consensus has been reached regarding SARS-CoV-2 infection [33,35,40,41]. A similar event may be possible for CD8 bystander cells. Many unanswered questions remain regarding B cells and the long-term memory response following vaccination [39]. Moreover, the effects of gender, obesity, and age remain unresolved regarding vaccine response [36].

The differences observed in the secretion of IFNγ and granzyme B are interesting (Figure 9A–D). The response is higher than expected, as observed in the analysis of subpopulations (Figure 9A,B). The increase in response to viral peptides compared to an inactivated virus (Figure 9B) raises the question of proper cell activation; however, there is no indication of any differences in cell response. Interestingly, there is no explanation for the difference in the granzyme B secretion between females and males with an inactivated virus (Figure 9D). The effect of the secreted cytokine and enzyme may be due to multiple cells, and this point should be further explored.

It is essential to note that this present report was conducted in an admixed population from Venezuela, which differs from other South American admixed populations. The response observed was lower than expected; conversely, it was similar to those previously reported [31,34,35,37]. Although herd immunity may play a crucial role in assessing the antiviral response in 2023, the effect observed here suggests that many questions remain about the memory response induced by the vaccine, which should be further studied.

## 5. Conclusions

The Sinopharm/BBIBP vaccine demonstrated the ability to elicit a memory response across various lymphocyte populations. Moreover, the immunity developed against the virus is sustained over time.

Gender and BMI incidence on immune response should be analyzed in a large cohort to determine the effect of these variables; however, the studies should be conducted longitudinally to avoid misinterpretations of data by a single analysis. Moreover, data on heterologous vaccines can also provide important information on the effect of the vaccine and memory response.

## 6. Limitations of the Study

One notable limitation of our study is the modest sample size (*n* = 52), which restricts the statistical power of robust subgroup analyses. Although we aimed to explore the cellular immune responses induced by the Sinopharm/BBIBP-CorV vaccine in a Venezuelan population, the small cohort size led to further compartmentalization by age, sex, BMI, and vaccine doses. This resulted in small subgroup sizes, potentially limiting our ability to detect differences in immune responses.

Although our analyses did not show significant differences in immune parameters between genders or BMI categories, these findings should be viewed cautiously due to the limited sample size, which may have affected our ability to detect variations seen in larger studies. Additionally, the number of vaccine doses may influence immune response, but our sample size did not permit a detailed comparison among these groups.

Our study focused on a Latin American admixed population, where few studies have assessed vaccine-induced cellular immunity. This context offers valuable insights due to its genetic diversity and distinct epidemiological dynamics. High vaccination rates and prior exposure to SARS-CoV-2 during the Omicron wave complicated the recruitment of a larger cohort and the inclusion of an unvaccinated control group.

## Figures and Tables

**Table 1:** Baseline characteristics of the patients by sex. ¥ Unpaired Student’s *t*-test; ^ Chis-square test with Yates’ correction.

Characteristics	Female (*n* = 30)	Male (*n* = 22)	*p*-Value
Age (mean)	44.37 (SD 9.141; CI 40.95–47.78)	49.05 (SD 8.415; CI 45.31–52.78)	0.0653 ¥
BMI (mean)	29.72 (SD 5.931; CI 27.50–31.93)	29.27 (SD 4.134; CI 27.44–31.10)	0.7627 ¥
BMI categories (*n*, %)			
Normal	7 (23%)	4 (18%)	0.9518 ^
Overweight	10 (34%)	9 (41%)	0.7879 ^
Obesity I	7 (23%)	7 (32%)	0.7150 ^
Obesity II/III	6 (20%)	2 (9%)	0.4913 ^
Sinopharm/BBIBP vaccine doses (*n*, %)			
4 doses	7 (23%)	4 (18%)	0.9518 ^
3 doses	16 (54%)	15 (68%)	0.4283 ^
2 doses	7 (23%)	3 (14%)	0.6027 ^
Comorbidities (*n*, %)			
Systemic arterial	4 (13%)	7 (32%)	0.245 ^
Hypertension.			
Diabetes Mellitus.	0	1 (5%)	0.8395 ^
COVID-19	17 (57%)	13 (59%)	0.9130 ^
SARS-CoV-2 Variants	Delta (2)	Delta (2)	
	Gamma (2)	Gamma (2)	

## Data Availability

The data of the study are available upon request.
